# Associations between primary healthcare and infant health outcomes: a cohort analysis of low-income mothers in Rio de Janeiro, Brazil

**DOI:** 10.1016/j.lana.2023.100519

**Published:** 2023-05-25

**Authors:** Thomas Hone, Jasper V. Been, Valeria Saraceni, Claudia Medina Coeli, Anete Trajman, Davide Rasella, Betina Durovni, Christopher Millett

**Affiliations:** a Public Health Policy Evaluation Unit, School of Public Health, Imperial College London, London, UK; b Division of Neonatology, Department of Neonatal and Paediatric Intensive Care, Department of Obstetrics and Gynaecology, and Department of Public Health, Erasmus MC Sophia Children’s Hospital, University Medical Centre Rotterdam, Rotterdam, the Netherlands; c Health Surveillance Branch, Secretaria Municipal de Saúde do Rio de Janeiro, Rio de Janeiro, Brazil; d Universidade Federal do Rio de Janeiro, Rio de Janeiro, Brazil; e Instituto de Saúde Coletiva, Universidade Federal da Bahia, Salvador, Brazil; f ISGlobal, Hospital Clínic— Universitat de Barcelona, Barcelona, Spain; g NOVA National School of Public Health, Public Health Research Centre, Comprehensive Health Research Center, CHRC, NOVA University Lisbon, Lisbon, Portugal

**Keywords:** Primary care, Infant health, Infant mortality, Brazil, Universal health coverage

## Abstract

**Background:**

Expanding primary healthcare to urban poor populations is a priority in many low-and middle-income countries and is essential to achieve universal health coverage (UHC). Between 2008 and 2016 the city of Rio de Janeiro undertook an ambitious programme to rapidly expand primary care to low-income areas through the family health strategy (FHS). Infant health impacts of this roll out are unknown. This study examines associations between maternal FHS utilisation and birth outcomes, neonatal and infant mortality.

**Methods:**

A cohort of 75,339 live births (January 2009–December 2014) to low-income mothers in Rio de Janeiro was linked to primary care, birth, hospital and death records. The relationship between maternal FHS use and infant health outcomes was assessed through logistic regression with inverse probability treatment weighting and regression adjustment. Socioeconomic inequalities in the associations between FHS use and outcomes were explored through interactions. Primary outcomes were neonatal and infant death. Thirteen secondary outcomes were also examined to explore other important health outcomes and potential mechanisms.

**Results:**

A total of 9002 (12.0%) infants were born to mothers in the cohort who used FHS services either before pregnancy or in the first two trimesters. There was a total of 527 neonatal and 893 infant deaths. Maternal FHS usage during the first two trimesters was associated with substantial reductions in neonatal [adjusted odds ratio (aOR): 0.527, 95% confidence interval (95% CI): 0.345; 0.806] and infant mortality (aOR: 0.672, 95% CI: 0.48; 0.924). Infants born to lower-income mothers and those without formal employment had larger reductions in neonatal and infant mortality associated with FHS use. Maternal FHS in the first two trimesters use was also associated with more antenatal care consultations and a lower risk of low birth weight and preterm birth.

**Interpretation:**

Expanding primary care to low-income populations in Rio de Janeiro was associated with improved infant health and health equity benefits.

**Funding:**

10.13039/501100002992DFID/MRC/10.13039/100010269Wellcome Trust/10.13039/501100000269ESRC.

**Research in context**

**Evidence before this study**

The World Health Organization (WHO) states that primary healthcare (PHC) is the main vehicle for achieving universal health coverage (UHC) and Sustainable Development Goal (SDG) target 3.2–reducing neonatal and early childhood mortality. However, many low-income urban populations lack access to quality primary care services. Limited knowledge on the impact of PHC on infant health and survival, especially in low-income urban populations, is hindering further investments in PHC.

EMBASE, MEDLINE, Global Health, HMIC (Health Management Information Consortium), and the Maternity & Infant Care Database via Ovid were searched in August 2022 for synonyms of “primary care”, “birth outcomes or infant health”, and “utilisation”. These were supplemented with internet searches (Google), searching of references in identified papers, and the authors’ own knowledge. Studies were restricted to those conducted in LMICs. Three recent systematic reviews highlight the relationship between antenatal care and reductions in neonatal and infant death, and improvements in birth outcomes (such a birth weight and risk of preterm birth). 32 studies examined the association between PHC services and infant health outcomes (covering Bangladesh, Bhutan, Brazil, The Gambia, Iraq, Mexico, Peru, South Africa, Tanzania, and Turkey), with a consistent pattern of increasing PHC use or coverage associated with health improvements. There is a wealth of evidence from Brazil, demonstrating expansion of PHC was associated with improvements in birth outcomes and reductions in infant mortality. However, most studies relied on cross-sectional surveys or ecological data, used methods which limit causal inference, and health inequalities were infrequently explored. There has also been limited research on whether integrating antenatal care (ANC) into PHC delivers health gains.

**Added value of this study**

This study uses linked individual-level health records within a cohort of livebirths (N = 75,339) born to low-income mothers to examine associations between PHC use and infant health outcomes in Rio de Janeiro. FHS use during the first two trimesters of pregnancy was associated with lower risks of neonatal or infant death, low birth weight or preterm birth and increases in ANC use. Lower-income mothers and those without formal employment had greater associated benefits from PHC use.

**Implications of all the available evidence**

PHC, including ANC delivered through PHC settings, is consistently associated with neonatal and infant mortality reductions–a finding supported by this robust, individual-level analyses. There is some evidence that more vulnerable and lower-income populations benefit more. PHC remains an essential platform for delivering health gains and is crucial for achieving UHC and the SDGs.

## Introduction

Sustainable Development Goal (SDG) target 3.2 seeks to end preventable deaths of newborns and children under five years of age by 2030. In 2020, there were an estimated 3.79 million infant deaths (i.e., within the first year of life), of which 63% (2.37 million) were neonatal deaths (i.e., within the first 28 days of life).^1^ In 2019, 60 countries were estimated to miss the SDG target of reducing neonatal mortality rates to 12 or fewer deaths per 1000 live births.^2^ Country-level metrics mask vast socioeconomic and geographical inequities within countries. For example, in 2017, within-country neonatal mortality varied by as much as 41.5 deaths per 1000 live births across local districts in 98 low- and middle-income countries (LMICs), with a mean within-country difference of 13.3 (121% relative difference).^3^ Low-income mothers and babies in urban LMIC environments may be particularly vulnerable as these settings are commonly characterised by high levels of poverty and a lack of public services.^4^

Strengthening primary healthcare (PHC) is central for making progress towards universal health coverage (UHC) and the SDGs.^5^^,^^6^ Stronger PHC is associated with financial protection against catastrophic health expenses^7^ and with better and more equitable health outcomes including reductions in neonatal and infant mortality.^8^, ^9^, ^10^, ^11^, ^12^ However, knowledge gaps remain. The impact of PHC on infant health outcomes is poorly explored in LMICs, including understanding the impacts on health inequalities. Individual-level healthcare data is rarely available for evaluating healthcare services–especially with linked outcome data such as mortality and birth outcomes. Addressing this knowledge gap is vital as primary care is often underfunded and underutilised in LMICs, where most (84%; 6.5 billion) of the world’s people live and where the largest burden of early life mortality (>98% neonatal deaths^1^) lies.

Brazil is an internationally important setting for generating evidence on PHC and child health. The country has large socioeconomic inequities, with over 42 million people (20% of the population) living on less than US$5.50 a day and 20% of the urban population living in slums or *favelas*.^13^ There are large inequalities in infant mortality across the country, with a rate of 12.5 infant deaths per 1000 live births in the richest 10% of municipalities compared to a rate of 26.4 in the poorest 10%.^14^ In 2015, the leading causes of death for under five years (of which 90% are infant deaths) were pre-term births, congenital abnormalities, birth trauma, neonatal infections and lower respiratory infections.^15^ In the city of Rio de Janeiro, one third of the population (2.5 million people) live in *favelas*,^16^ where infant mortality rates are five times higher than in other parts of the city.^17^ Brazil has had a strong commitment to PHC through the world-renowned *Estratégia de Saúde da Família* (Family Health Strategy; FHS)^18^ (Panel 1), and a range of studies demonstrate that nationwide expansion of the FHS has been associated with improved health outcomes, including reductions in infant mortality and improved birth outcomes.^10^^,^^19^, ^20^, ^21^, ^22^, ^23^, ^24^, ^25^, ^26^, ^27^, ^28^, ^29^ However, nearly all of these studies rely on aggregate, ecological data to analyse FHS expansion, limiting causal inference and the opportunity to study inequalities in detail.

Panel 1 **The family health strategy (FHS) in Brazil and Rio de Janeiro.**

The 1988 constitution of Brazil guaranteed a right to health for all Brazilians and charged the government of Brazil with the responsibility for delivering healthcare to meet this obligation. This led to the creation of the unified health system (Sistema Único de Saúde; SUS) in 1990. The FHS began as a community workers primary care programme in the 1990s–aiming to expand basic services to underserved regions in the North and Northeast of the country. The FHS was later adopted nationwide and expanded to include higher-skilled health professionals and a comprehensive package of services.^18^

Today, the FHS is a multidisciplinary model of PHC including doctors, nurses and community health agents where each team covers ∼1000 local families and provides services including health promotion, health education, risk factor management, prevention of diseases, home visits, acute care, and referral.^30^ Services are free to use and cover locally registered populations. A large complementary private system operates separately to SUS for higher-income populations (covering 25–30% of the population nationwide), meaning the FHS generally serves low-income populations and those with greater health needs.^18^

In Rio de Janeiro, PHC coverage was rapidly expanded beginning in 2008, with prioritised expansion in poorer areas. In 2008, the municipal government of the city of Rio de Janeiro spent the least on healthcare of any state capital, with over 80% of spending directed towards hospitals.^31^^,^^32^ FHS coverage was low at around 7%, and where services were available, many teams (∼40%) lacked doctors.^31^ Low-income populations had to use hospital emergency rooms, costly private care, or philanthropic clinics.^33^, ^34^, ^35^ High levels of infant mortality were also an important rationale for the expansion of FHS within the city,^32^ and by 2016, over 50% of the city’s population were covered by the FHS. FHS services in the city are based closely to the national FHS model with multidisciplinary teams and defined catchment areas. However there were local adaptations with a focus on quality, coordination, and efficiency, and the health of the urban poor,^31^ with use of clinical guidelines, training and workshops, attention to team management, and quality monitoring.^31^^,^^32^ Additionally, investments were made in clinics, where multiple FHS teams are co-located, to install radiological services, ultrasound, and equipment for minor surgery.^36^

This study uses a cohort of 75,339 live births to low-income mothers with linked welfare-claimant data, PHC registration records and mortality records to examine associations between maternal FHS utilisation and neonatal and infant mortality. We also explore effects on antenatal care (ANC), birth outcomes, and infant hospitalisations to assess potential mechanisms and other important health outcomes. The city of Rio de Janeiro was chosen for this research given the city-wide expansion of the FHS programme and investments in frontline services (Panel 1) during the study period, and the availability of linked data on a cohort of low-income individual covering roughly 25% of the city population^37^^,^^38^—a unique situation the country. Given the known benefits of PHC on infant health, we hypothesize that FHS utilisation may be associated with improved birth outcomes and infant health.

## Methods

### Study design

The cohort used for this study includes all births to low-income mothers in the city of Rio de Janeiro over the period 1st January 2009–31st December 2014, i.e., during FHS expansion. Associations between maternal FHS usage, birth outcomes and child mortality were analysed through regression modelling. Doubly robust inverse probability treatment weighting and regression adjustment (IPTW-RA) were employed to strengthen causal inference. This is a widely used approach to reduce bias from potential differences in control (FHS non-users) and ‘treated’ (FHS users) individuals in observational studies.^37^^,^^39^

### Data sources and linkage

Data for the cohort is based on linkage of administrative, health and mortality databases in individuals registered with *Cadastro Único* for government welfare. *Cadastro Único* is a national administrative database which includes approximately 25% of the population of the city of Rio de Janeiro who chose to apply for government welfare and contains individual- and household-level socioeconomic and demographic data. We obtained the 2015 *Cadastro Único* which included all individuals registered up until 31st December 2014. This original cohort contained 1,762,905 individuals, of which 95,093 (5.4%) were excluded due to duplicate *Cadastro Único* records, invalid registration, and if individuals that died before the start of the cohort. The mean observation period was 5.24 years. To obtain a cohort of infants born to mothers registered with the *Cadastro Único*, data linkage was undertaken. First, *Cadastro Único* was linked to SINASC (*Sistema de Informações sobre Nascidos Vivos*), Brazil’s national live birth database containing birth certificate data. Coverage and data quality for the city of Rio de Janeiro is high (e.g., 99.7% of all births registered).^38^^,^^40^ We identified all live births to mothers within the *Cadastro Único* including infants already registered in *Cadastro Único* and those who were not. Stillbirth records were not available for linkage. A total of 76,246 live births were identified, of which 846 birth records (SINASC) were identified as duplicate records and 61 had missing data on sex or race (1.2% excluded).

This live birth cohort was linked to additional databases: i) mother’s FHS electronic health records; ii) neonatal and infant mortality records for 2009–2015; and iii) both infant and mother’s public hospitalisation records. Only infants within the *Cadastro Único* were linked to hospitalisation records as infant names are not recorded on birth certificates, inhibiting linkage. All datasets were linked via a combination of deterministic and probabilistic approaches (as published elsewhere^38^) which involved matching name, date of birth and tax numbers using deterministic linkage, phonetic matching, and Levenshtein distance matching.

The most relevant variables for child health were extracted from the datasets. From *Cadastro Único*: mother’s race/ethnicity, mother’s disability, mother’s employment status, household total income, household’s access to piped water, child labour in the household, formal employment in the household, number of household inhabitants, number of bedrooms in household, household expenditure on medicines, household *Bolsa Familia* payments (conditional welfare programme), *bairro* (neighbourhood) of residence, and mother’s date of joining the *Cadastro Único*. From birth certificates: infant’s sex, infant’s month and year of birth, infant’s birth weight, mode of delivery, location of delivery, gestational age, number of ANC consultations (recorded categorically on the birth records), if the birth was single or multiple, mother’s age, mother’s marital status, mother’s educational attainment, if mother has other children, and if mother had lost other children. From mortality records: infant’s date of death. From hospitalisation records: dates of admissions for both mothers and infants. From FHS records: the dates of mother’s consultations with FHS clinics.

### Outcomes and exposure variables

The two primary outcomes were: neonatal death (within 28 days of birth) and infant death (within one year of birth). Thirteen secondary outcomes were also examined to explore other important health outcomes and potential mechanisms of action covering prenatal care, delivery, birth outcomes, and hospital admission. The first nine secondary outcomes were binary variables–specifically: i) no ANC received; ii) 1–3 ANC consultations received; iii) 4–6 ANC consultations; iv) 7+ ANC consultations; v) caesarean delivery; vi) preterm birth (birth at less than 37-week gestation); vii) very preterm birth (less than 32-week gestation); viii) low birth weight (less than 2500 g); and ix) very low birth weight (less than 1500 g). We explore ANC as a secondary outcome measure to explore potential mechanisms of action. Four additional secondary outcomes were counts of hospital admissions: vii) hospitalisations of mother during pregnancy; viii) hospitalisations of mother 7–365 days since delivery; ix) hospitalisations of infant 7 days to 3 months since birth; and x) hospitalisations of infant during months 4–12 after birth. Infant hospitalisation counts were only available in the sub-set of infants that were registered within the *Cadastro Único*.

Two main exposure variables of interest were generated related to mother’s FHS utilisation before and during pregnancy. These were *i*) any FHS utilisation prior to pregnancy; and *ii*) any FHS utilisation (for any reason) in the first two trimesters of pregnancy. Analyses did not focus on third trimester FHS usage as the most health benefits and risk reductions come from healthcare use during the first two trimesters.^41^ When examining the effect of FHS usage on post-birth hospitalisations (the outcomes), an additional exposure was generated–mother or child FHS consultation in first three months after birth.

Other variables used in the analyses were grouped at household-, mother-, and child-levels. Household variables were: household per capita income quintile; household inhabitants per bedroom (two or fewer; two-three; three-four; more than four); household per capita monthly expenditure on medicine (none; Brazilian Reals (R$)0–50; more than R$50); if there was formal employment in the household; if the household received Bolsa Familia; household with piped water; and child labour in the household. Mother variables were: self-identified race/ethnicity (white; black; pardo (brown); Asian, indigenous or other); marital status (single; married or civil union; widow, separated or other); age category (less than 17 years old; 17–19; 20–24; 25–29; 30–34; 35–39; 40–44; 45 years or more), educational attainment (less than three years of schooling; 4–7; 8–11; 12 or more years); self-reported disability (yes; no); employment status (yes; no); if the mother had other children (yes; no); and if the mother had other children who died (yes; no). Infant variables were: month of birth; year of birth; type of birth (single; twins; triplets or more); and sex (male; female).

Additional variables that were derived for selected analyses: mother’s neighbourhood of residence; number of hospitalisations the mother had prior to pregnancy (2009–2015); if the birth was in a private hospital; and mother’s year of cohort entry.

### Analyses

Weights for IPTW were constructed by conducting logistic regression on the likelihood of mother’s FHS use at any point before the third trimester of pregnancy (including consultations pre-pregnancy). The model was adjusted for all household- and mother-level variables noted above in addition to mother’s year of cohort entry, neighbourhood of residence, and pre-pregnancy hospitalisations. These variables aimed to capture factors predicting FHS usage in pregnancy including deprivation, maternal characteristics, neighbourhood factors, and earlier health needs. The model selection process initially included all relevant covariates with potential confounding effects that were available in the data and collinearity checked through variance inflation factors (VIFs). Given the low VIFs, no covariates were excluded. Predicted probabilities were used to generate stabilised weights for IPTW. Stabilised weights were used to weight all regression models. Doubly-robust estimators were obtained by using both IPTW and adjustment for potential confounders through regression models.

The association between maternal FHS usage and our outcomes were assessed through multi-level regression models (See Supplementary material for estimating equations and more detail). All primary outcomes and binary secondary outcomes were modelled with logistic regression (with adjusted odds ratios (aOR) reported), whilst secondary count outcomes (hospitalisations) were modelled with Poisson regression (adjusted rate ratios (aRR)). Multi-level approaches accounted for the clustered/hierarchical nature of the observations (births clustered by mothers). A random intercept model was chosen with intercepts at the mother level. Separate models were repeated for each specification of three FHS usage variables defined above. Models on post-birth hospitalisations were also adjusted for FHS utilisation by the mother in the first three months after delivery. Models analysing infant hospitalisations only analysed the subsample of infants described above. Models were adjusted for all household-, mother-, and infant-level variables noted above. Standard errors were clustered by mothers.

Heterogeneity in the association between FHS use and outcomes was assessed through interactions. FHS usage in the first two trimesters (binary) was interacted with key socioeconomic and demographic variables, and also a categorical variable denoting ANC use. Three measures of socioeconomic status were used: i) household income per capita quintile; ii) maternal race/ethnicity; and iii) formal employment status of the household. This allowed identification of inequalities in associations between FHS use and outcomes by socioeconomic groups.

### Sensitivity analyses

Analyses only examined FHS utilisation during the first two trimesters as this is the period when most health benefits and risk reductions are accrued.^41^ Models for all full-term outcomes were repeated examining only FHS use in third trimester to assess potential biases. Models were also repeated only including the first birth per mother (n = 66,034) to evaluate potential biases from multiple births per mother.

### Ethical approval

Approval for this study was obtained from Imperial College London and the Brazilian National Commission for Ethics in Research (*Comissão Nacional de Ética em Pesquisa* (CONEP))–number 2.689.528.

The authors had full access to all anonymised databases employed in this analysis. Identifiable datasets for linkage were securely held by co-author (C Medina Coeli) for carrying out linkages and the generation of linkage keys to link the anonymised datasets.

### Role of the funding source

This study was supported by the UK’s Joint Health Systems Research Initiative (DFID/MRC/Wellcome Trust/ESRC) grant number MR/P014593/1. This funder had no role in the study design, in the collection, analysis, and interpretation of data, in the writing of the report, or in the decision to submit the paper for publication.

## Results

There were 75,339 live births to 66,034 mothers in the period 1st January 2009–31st December 2014 (Table 1). Mean births per mother was 1.14 with a maximum of five. 9002 births (12.0%) were to mothers who used FHS services before the third trimester of the pregnancy (7926 mothers used FHS during the 1st two trimesters and 3460 before pregnancy). No infants born in 2009 had mothers who used FHS services, but this increased to 33.3% in 2014. Mothers who were older, not married, had no previous children, had been hospitalised before, had 4–11 years of education, and lived in households with formal employment, that were less crowded and were in income quintiles 2–4 were more likely to use FHS (Supplementary material).

**Table 1:** Characteristics and outcomes of the cohort.

	Overall		No FHS use		Any maternal FHS consultation before pregnancy		Any maternal FHS consultation 1st or 2nd trimester	
	N	Unweighted %	N	IPTW weighted %	N	IPTW weighted %	N	IPTW weighted %
**Primary outcomes**								
Neonatal deaths	527	0.7	478	0.7	46	1.5	71	0.9
Infant deaths	893	1.2	801	1.2	30	1.0	36	0.4
**Secondary outcomes**								
No ANC	2030	2.7	1953	3.0	35	1.1	59	0.8
1–3 ANC consultations	5647	7.5	5351	8.3	153	4.8	194	2.6
4–6 ANC consultations	22,094	29.3	20,038	31.1	776	22.6	1754	22.7
7+ ANC consultations	43,602	57.9	37,180	57.7	2431	71.5	5789	73.9
Caesarean births	31,725	42.1	28,058	41.8	1471	45.9	3168	44.2
Very preterm births	1340	1.8	1218	1.8	65	1.9	92	1.1
Preterm births	8178	10.9	7253	11.0	387	11.4	772	9.4
Very low birth weight (<1500 g)	1226	1.6	1120	1.7	50	1.5	85	1.2
Low birth weight (<2500 g)	7184	9.5	6468	9.7	288	8.7	607	7.5
Mother hospitalised during pregnancy	4525	6.0	3711	5.8	335	8.9	729	8.4
Mother hospitalised 7-days to 12 months after delivery	1366	1.8	1148	1.8	93	2.4	197	2.0
Infant hospitalised 7-days to 3 months after birth^a^	794	1.1	697	1.5	37	2.9	88	2.3
Infant hospitalised 4–12 months after birth^a^	1480	2.0	1315	2.7	62	3.9	133	3.1
**Infant-level variables**								
Sex								
Male	38,870	51.6	34,209	51.6	1774	51.5	4086	51.6
Female	36,469	48.4	32,128	48.4	1686	48.5	3840	48.5
Birth plurality								
Single	73,492	97.5	64,637	97.5	3422	98.5	7800	98.4
Twins	1816	2.4	1671	2.5	37	1.4	125	1.6
Triplet or more	31	0.0	29	0.0	1	0.0	1	0.0
Year of birth								
2009	10,206	13.5	10,206	15.0	0	0.0	0	0.0
2010	10,827	14.4	10,824	15.9	1	0.0	3	0.0
2011	13,405	17.8	13,252	20.1	4	0.2	152	1.9
2012	14,328	19.0	12,850	19.5	283	8.2	1388	17.5
2013	13,788	18.3	10,681	16.4	1141	32.7	2746	34.7
2014	12,785	17.0	8524	13.1	2031	58.9	3637	45.9
**Mother-level variables**								
Maternal age								
<17 years	8506	11.3	7416	11.3	420	11.2	969	12.2
18–19 years	7773	10.3	6783	10.3	378	10.6	854	10.8
20–24 years	19,588	26.0	17,365	26.0	834	24.5	1957	24.7
25–29 years	17,337	23.0	15,297	23.0	785	23.4	1809	22.8
30–34 years	13,415	17.8	11,804	17.8	602	17.7	1421	17.9
35–39 years	6770	9.0	5959	9.0	339	9.7	708	8.9
40–44 years	1838	2.4	1615	2.4	97	2.8	194	2.5
45–50 years	112	0.1	98	0.2	5	0.2	14	0.2
Mother's marital status								
Single	62,559	83.0	54,892	83.0	2999	85.0	6745	85.1
Married/Civil Union	11,554	15.3	10,371	15.4	405	13.3	1053	13.3
Widow/Separated/Other	1226	1.6	1074	1.6	56	1.8	128	1.6
Maternal educational attainment								
Less than 3 years	3342	4.4	3013	4.5	124	4.1	294	3.7
4–7 years	23,644	31.4	21,213	31.4	959	31.0	2132	26.9
8–11 years	44,184	58.6	38,170	58.6	2294	61.5	5298	66.8
12+ years	4169	5.5	3941	5.6	83	3.4	202	2.6
Mother has other children	48,938	65.0	43,347	65.0	2244	68.6	4831	61.0
If mother has lost other children (incl abortion)	22,297	29.6	19,885	29.7	968	29.7	2102	26.5
Maternal race/Ethnicity								
White	20,289	26.9	17,904	26.9	887	25.9	2106	26.6
Black	15,346	20.4	13,618	20.4	669	20.3	1514	19.1
Parda	39,273	52.1	34,386	52.1	1904	53.9	4304	54.3
Asian or indigenous or other	431	0.6	429	0.6	0	0.0	2	0.0
Mother has disability	980	1.3	886	1.3	48	1.4	75	1.0
Mother employed	34,943	46.4	30,828	46.4	1574	46.5	3623	45.7
**Household-level variables**								
Child labour in household	695	0.9	606	0.9	37	1.1	77	1.0
If household has piped water	74,043	98.3	65,169	98.3	3409	98.2	7809	98.5
Bolsa Familia recipient household	66,039	87.7	58,124	87.6	3090	89.4	6950	87.7
Inhabitants per bedroom								
Less than two	24,902	33.1	21,775	33.1	1168	32.6	2780	35.1
Two to three	22,818	30.3	20,149	30.3	1046	30.1	2353	29.7
Three to four	14,761	19.6	13,004	19.6	676	20.1	1538	19.4
Four or more	12,858	17.1	11,409	17.0	570	17.2	1255	15.8
Household expenditure on medicines								
None	61,234	81.3	53,907	81.3	2802	80.3	6455	81.4
0–R$50 per month	10,506	13.9	9229	13.9	509	15.0	1116	14.1
More than R$50 per month	3599	4.8	3201	4.8	149	4.7	355	4.5
Formal employment in household	16,265	21.6	14,322	21.6	725	20.6	1732	21.9
Household per capita income quintile								
Q1 (lowest)	15,179	20.1	13,507	20.2	669	20.6	1449	18.3
Q2	14,955	19.9	13,034	19.8	788	21.1	1674	21.1
Q3	15,105	20.0	13,190	20.0	753	21.7	1692	21.4
Q4	15,031	20.0	13,272	20.0	654	19.5	1546	19.5
Q5 (highest)	15,069	20.0	13,334	20.0	596	17.1	1565	19.8
N (Observations)	75,339		66,337		3460		7926	

ANC–antenatal care; FHS–family health strategy; IPTW–inverse probability of treatment weighting.

a Calculated out of a subset of infants with linked hospitalisation records (N = 52,390).

Mothers of 59.4% (43,602) births received seven or more ANC consultations throughout their pregnancy, 42.1% (31,725) of births were by caesarean, 1.7% (1340) were very preterm, 10.9% (8178) were preterm, 1.6% (1226) were very low birth weight, and 9.5% (7184) were low birth weight. 0.7% (527) died within 28 days of birth (neonatal death), and 1.2% (893) died within one year of life (infant death). There were 4525 hospitalisations of mothers during pregnancy (6.0% of births) and 1366 (1.8%) hospitalisations of mothers during 12 months post-delivery. For infant hospitalisations, 52,390 infants were included in the subgroup analysis (only *Cadastro Único* registered infants), of whom 794 were hospitalised 7 days to 3 months post birth (1.5%) and 1480 were hospitalised 4–12 months after birth (2.8%).

In IPTW-RA multi-level logistic regression models, any FHS usage during the first two trimesters of pregnancy was associated with a lower likelihood of neonatal [aOR: 0.527, 95% confidence interval (95% CI): 0.345–0.806] and infant death (aOR: 0.672, 95% CI: 0.488–0.924) (Table 2). In post-regression modelling, these effect estimates translated into an averted 254 infant deaths (or which 218 were neonatal deaths) had all infants been born to mothers that used FHS during the first two trimesters of pregnancy. There was no association between maternal FHS use before pregnancy and neonatal or infant death.

**Table 2:** Results from IPTW-RA multilevel logistic regression models on neonatal and infant death, ANC consultations, and birth outcomes.

	Any maternal FHS consultation before pregnancy		Any maternal FHS consultation 1st or 2nd trimester	
	aOR	95% CI	aOR	95% CI
**Primary outcomes**				
Neonatal death	1.397	0.841, 2.321	0.527∗∗	0.345, 0.806
Infant death	1.169	0.789, 1.734	0.672∗	0.488, 0.924
**Secondary outcomes**				
No ANC	0.490∗∗	0.313, 0.768	0.334∗∗∗	0.236, 0.471
1–3 ANC cons	0.612∗∗∗	0.492, 0.762	0.278∗∗∗	0.230, 0.336
4–6 ANC cons	0.682∗∗∗	0.608, 0.764	0.668∗∗∗	0.617, 0.723
7+ ANC cons	1.809∗∗∗	1.597, 2.050	2.262∗∗∗	2.069, 2.473
Caesarean birth	1.641∗∗∗	1.343, 2.005	1.245∗∗	1.084, 1.430
Very preterm birth	0.946	0.647, 1.383	0.476∗∗∗	0.350, 0.645
Preterm birth	0.975	0.812, 1.170	0.661∗∗∗	0.578, 0.757
Very low birth weight	0.899	0.594, 1.361	0.631∗∗	0.461, 0.863
Low birth weight	0.914	0.743, 1.124	0.690∗∗∗	0.594, 0.800

Each coefficient is from a separate logistic regression model. All models were adjusted for: month and year of birth; type of birth (single; twins; triplets or more); infant’s sex; mother’s self-identified race/ethnicity; mother’s marital status; mother’s age; mother’s educational attainment; mother’s disability; mother’s employment status; if the mother has other children; if the mother had other children who died; household income quintile; household inhabitants per bedroom; household per capita monthly expenditure on medicine; household formal employment; household Bolsa Familia receipt; household access to piped water; and if there was child labour in the household. Standard errors clustered by mothers. Models weighted by IPTW.

ANC–antenatal care; FHS–family health strategy; IPTW-RA–inverse probability of treatment weighting with regression adjustment; aOR—adjusted odds ratio; 95% CI–95% confidence interval.

∗p < 0.05, ∗∗p < 0.01, ∗∗∗p < 0.001.

For secondary outcomes, maternal FHS utilisation both prior to pregnancy and during the first two trimesters were associated with reductions in no ANC, 1–3, and 4–6 ANC consultations with a concomitant increase in likelihood of having 7 or more ANC consultations (Table 2). For example, any maternal FHS consultation in the first two trimesters was associated with 67% lower likelihood of having no ANC (aOR: 0.334, 95% CI: 0.236–0.471) and 2.3 times increase in the likelihood of having seven or more ANC consultations (aOR: 2.262, 95% CI: 2.069–2.473). Both pre-pregnancy and first two trimester FHS use were associated with higher likelihood of caesarean delivery (aOR: 1.641, 95% CI: 1.343–2.005; and aOR: 1.245, 95% CI: 1.084–1.430). Any FHS use in the first two trimesters was associated with a 52% lower likelihood of very preterm birth (aOR: 0.476, 95% CI: 0.350–0.645), a 34% lower likelihood of preterm birth (aOR: 0.661, 95% CI: 0.578–0.757), a 37% lower likelihood of a very low weight birth (aOR: 0.631, 95% CI: 0.461–0.863), and a 31% lower likelihood of low weight birth (aOR: 0.690, 95% CI: 0.594–0.800). Pre-pregnancy FHS usage was not associated with any birth outcomes.

FHS usage during pregnancy or the first two trimesters was not associated a risk of hospitalisation for either infants or mothers. However, the exceptions were maternal FHS use in the first two trimesters, which was associated with 23% lower risk of admission of infants 4–12 months post birth (aRR: 0.771, 95% CI: 0.610–0.974), and maternal or infant FHS use in the three months since birth which was associated with a 21% lower risk of admission of infants 7 days to three months post birth (aRR: 0.794, 95% CI: 0.633–0.996) (Table 3).

**Table 3:** Results from multilevel Poisson regression models on hospital admissions.

	Any maternal FHS consultation before pregnancy		Any maternal FHS consultation 1st or 2nd trimester		Any maternal or infant FHS consultations in 3 months since birth	
	aRR	95% CI	aRR	95% CI	aRR	95% CI
Maternal admissions during pregnancy	1.015	0.878, 1.172	0.931	0.840, 1.032	–	–
Maternal admissions 7 days-12 months post delivery	0.983	0.746, 1.296	0.835	0.689, 1.013	0.956	0.799, 1.144
Infant admissions 7 days-3 months post birth	1.278	0.825, 1.978	1.026	0.776, 1.356	0.794∗	0.633, 0.996
Infant admissions 4–12 months post birth	1.019	0.712, 1.458	0.771∗	0.610, 0.974	0.949	0.797, 1.130

Each coefficient is from a separate Poisson regression model. All models were adjusted for: month and year of birth; type of birth (single; twins; triplets or more); infant’s sex; mother’s self-identified race/ethnicity; mother’s marital status; mother’s age; mother’s educational attainment; mother’s disability; mother’s employment status; if the mother has other children; if the mother had other children who died; household income quintile; household inhabitants per bedroom; household per capita monthly expenditure on medicine; household formal employment; household Bolsa Familia receipt; household access to piped water; and if there was child labour in the household. Standard errors clustered by mothers. Models weighted by IPTW.

FHS–family health strategy; IPTW-RA–inverse probability of treatment weighting with regression adjustment; aRR–adjusted Rate Ratio; 95% CI–95% confidence interval.

∗p < 0.05.

**Fig. 1:**
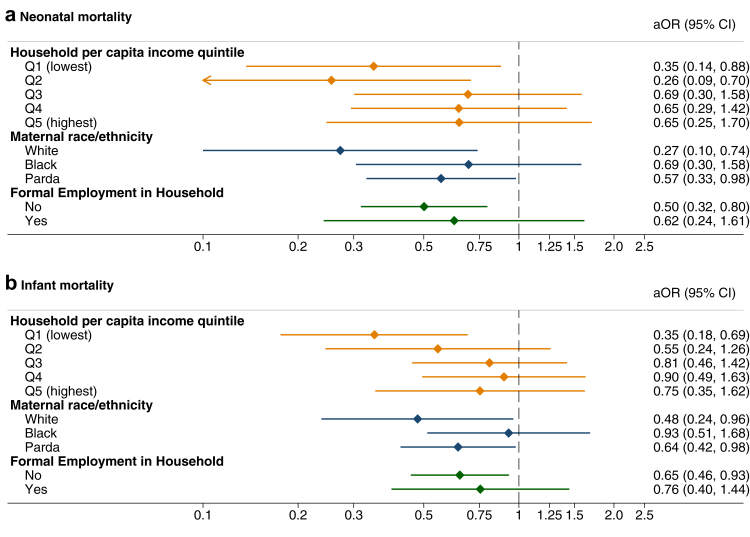
**Inequalities in association between FHS use during pregnancy and (a) neonatal and (b) infant mortality by socioeconomic groups**. Each plotted coefficient is from a separate regression model with an interaction between variable indicating a FHS consultation during first two trimesters of pregnancy and one socioeconomic variable (income, marital status or race/ethnicity). All models were adjusted for: month and year of birth; type of birth (single; twins; triplets or more); infant’s sex; mother’s self-identified race/ethnicity; mother’s marital status; mother’s age; mother’s educational attainment; mother’s disability; mother’s employment status; if the mother has other children; if the mother had other children who died; household income quintile; household inhabitants per bedroom; household per capita monthly expenditure on medicine; household formal employment; household Bolsa Familia receipt; household access to piped water; and if there was child labour in the household. Standard errors clustered by mothers. Models weighted by IPTW. The coefficients for the race/ethnicity group Asian or Indigenous or other were not plotted due to small numbers and very large confidence intervals.

Although imprecisely estimated with wide confidence intervals due to small numbers, there was evidence of heterogeneity in the associations between FHS usage in the first two trimesters and neonatal and infant mortality (Fig. 1). Mothers in the lowest two income quintiles, of white race/ethnicity, or without formal employment in the household had larger associated reductions in infant and neonatal mortality that other demographic and socioeconomic groups. Lower income mothers, those of Black race/ethnicity, and without formal employment had greater associated increases in seven or more ANC consultations with FHS use (Supplementary material).

### Sensitivity analyses

Examining FHS use in the third trimester of pregnancy found FHS use was not associated with neonatal or infant mortality, however improvements in ANC usage and birth outcomes were found (Supplementary material). The results including only first births per mother, yielding highly comparable results and effect sizes across all outcomes Supplementary material.

## Discussion

Infants born to mothers who utilised FHS services during the first two trimesters of pregnancy had large associated improvements in child health outcomes and survival. This included reductions in likelihood of neonatal and infant mortality, of low or very low birth weight, and of preterm or very preterm birth. Lower-income mothers appeared to benefit more from FHS usage than higher-income mothers.

These findings underscore the vital importance of PHC during pregnancy and align with previous research on the infant and wider health benefits of PHC in Brazil^10^^,^^20^^,^^22^^,^^42^^,^^43^ and other LMICs.^8^^,^^9^^,^^44^, ^45^, ^46^ Key facets of PHC services may contribute to improved infant health. For example, health education of expectant mothers and their families is important for reducing risk of neonatal death.^47^ Facilitating access to ANC appears to be an important mechanism by which the FHS can also deliver health benefits, as increased ANC use has been associated with reduced risk of low birth weight in LMICs.^30^^,^^31^^,^^38^ Health education, prevention, screening, and referral are key features of the FHS and likely contributed to the positive infant survival and health benefits identified. Furthermore, more appropriately referring high-risk mothers to maternity services, as part of ANC service changes with FHS expansion,^48^ may have delivered health gains to the most at-risk mothers. Notably, FHS use was also associated with increased chances of caesarean delivery. This could be due to increased use of FHS and referral of high-risk mothers, but given the extremely high rates of caesarean in Brazil (57% in 2021^49^) and limited clinical reason for most caesarean deliveries in the country,^50^ this finding could be indicative of problems with the model of care.

A second key set of findings relates to heterogeneity in the effects of FHS use. Generally, children born to more deprived mothers (e.g., lower income or no formal employment in household) had larger increases in ANC use and larger relative reductions in risk of death associated with FHS use than children born to more affluent mothers. This aligns with the inequality-reducing aspects of the FHS identified in Brazil,^25^^,^^37^^,^^43^^,^^51^ and also evidence on expanding access to PHC more generally in LMICs.^6^^,^^52^^,^^53^ For example, expanding access to PHC has been associated with reductions in health inequalities in Colombia.^54^ The inequality-reducing aspects of FHS in Rio may be due to the pro-poor direction of the expansion of the FHS and increased access to the FHS for previously underserved populations.^31^ PHC, in general, can reduce inequalities as it is more effective than specialist care in addressing population health needs and increasing access to healthcare.^55^

There was a notable finding that babies born to Black mothers did not experience the same improvements in birth and health outcomes from maternal FHS use as white or pardo mothers, except for increases in ANC use, where Black mothers differentially benefitted. These findings may stem from underlying racial/ethnic injustices in Brazil where individuals of darker skin colour have greater health needs^56^ but are disadvantaged and discriminated against throughout society resulting in lower access to key public services.^57^ Furthermore, where they do have access to healthcare, medical racism may systematically impact the care Black individuals receive. In this study, the FHS may be facilitating access to ANC for Black mothers at relatively greater rates than white or pardo mothers. However, the lack of association between FHS use and birth outcomes or mortality for babies born to Black mothers suggests specific barriers to improving the health of these infants persist along the FHS-ANC-delivery-postpartum care continuum, which warrants further attention.

This study has limitations. The study is observational, and despite some randomness in the roll-out of the FHS in Rio de Janeiro and the use of IPTW-RA, selection bias relating to FHS use may remain. There could be unobserved factors associated with ANC use, FHS use, and health outcomes such as care seeking behaviours, health knowledge, self-care, and other socioeconomic factors that remained unaccounted for in our analysis. However, a large number of covariates were included in the models which likely acted, at least partially, as proxies for these factors. There are limitations from the data used. Unfortunately, updated data is not available due to changes in the electronic health records in Rio de Janeiro, limiting inference for the present day. Data on the timing of receipt of social welfare was also not available. The quality of delivery services in hospital was also not known and may bias the findings (i.e., with mothers of lower income and socioeconomic status more likely to receive lower-quality care), although given the low-income focus of the whole cohort the variability in quality may not have been as large relative to the city of Rio de Janeiro as a whole. ANC use and birth weight were encoded categorically on birth certificates precluding the ability to analyse the timing of ANC care or to calculate birth weight centile. Some births (1.2%) were excluded due to duplicates or missing data. It was not known the reasons why women attended ANC and if a problematic pregnancy led to more ANC visits, this may have biased the results towards the null. It was also not possible in analyses to account for lower levels of ANC use for women who delivered preterm, but who may have higher levels of risk, which may have biased the findings. There may also be overlap between ANC and FHS usage, meaning the results on ANC use should be interpreted with caution. Despite the size of the cohort, small numbers precluded further inequality analyses and wide confidence intervals were present for many analyses. Data on stillbirths were not available and this remains an area for future research. Infant hospitalisation data was only available for infants in *Cadastro Unico*, and so the highest risk infants (who died before registering *Cadastro Único*) were not captured, potentially underestimating the associations found. Lastly, we tested multiple outcomes and although there could be concerns over multiple outcome testing, conservative Bonferroni adjustment would still have produced statistically significant findings for key outcomes.

The study strengthens the evidence base supporting the benefits of the FHS systems in Brazil and re-enforces the importance of PHC-orientated health systems for all countries, especially for low-income populations. The infant survival and health benefits and reductions in inequalities in these outcomes identified serve as a timely reminder to policymakers of the need to invest in PHC. This evidence builds on the broad knowledge of the importance of PHC for achieving UHC and the SDGs.^5^^,^^6^ Further evidence is needed on the topic, including investigating better adjustment for high-risk pregnancy, and understanding whether these women and babies benefit from FHS use differently. Further investigation into the reasons for maternal PHC utilisation would be valuable to understand which specific interventions are potentially delivering health gains, and better quantification of inequalities in accrued benefits would be useful. Further robust studies which use individual-level data are needed to further generalise these findings to other settings, including rural populations, vulnerable communities, and poorer and less economically strong urban areas. There remains a need to generate further causal evidence on the impacts of PHC in other LMICs.

### Conclusions

The expansion of PHC in Rio de Janeiro between 2008 and 2015 to poor urban populations was associated with improvements in infant survival and health outcomes, especially for lower-income mothers and those without formal employment. This evidence contributes to the understanding that PHC is an essential platform for strengthening health systems and delivering health gains to vulnerable populations in LMICs.

## Contributors

This study was conceived by BD, CM, TH, and DR. Data acquisition and linkage were undertaken by VS and CMC. VS, CMC, and TH accessed and verified the data. TH undertook the analyses with inputs from AT, JB, and CM. TH wrote the first draft of the paper with inputs, revisions, and edits from all authors.

## Data sharing statement

The datasets generated and analysed during the current study are not publicly available due confidentiality of the linked data. They are available from the corresponding author on reasonable request and following approval from the Brazilian National Commission for Ethics in Research (Comissão Nacional de Ética em Pesquisa (CONEP)) and the Municipal government of Rio de Janeiro.

## Declaration of interests

BD was Undersecretary of Health Promotion, Surveillance, and Primary Care at the Secretaria Municipal de Saúde, Rio de Janeiro when this project was conceived. VS is a Coordinator of Health Situation Analysis in the Health Surveillance Department, at the Secretaria Municipal de Saúde, Rio de Janeiro. All other authors declare they have no competing interests.

## Appendix ASupplementary data

Supplementary material

Abstract in portuguese
