# Supplementary material for: Associations between primary healthcare and infant health outcomes: a cohort analysis of low-income mothers in Rio de Janeiro, Brazil
Source: Lancet Reg Health Am. 2023 May 25;22:100519. doi: 10.1016/j.lana.2023.100519 (PMC10238835; doi:10.1016/j.lana.2023.100519)
Supplement: Supplementary material [file mmc1.docx]

**SUPPLEMENTARY MATERIAL**

**Text S1 – Estimating equations**

The weights for IPTW are estimated using a logistic regression model with the specification:

$$\Pr\left( y=1 \right)=F(\beta_{0}+ \beta_{n}x_{n})$$

Where $y$ refers to the outcome (any maternal FHS utilisation before the third trimester of pregnancy), $\beta_{0}$ refers to the constant, and $x_{n}$ refers to mother- and household-level variables with their respective coefficients $\beta_{n}$. *F(z) = e^z^ / (1 + e^z^)* is the cumulative logistic distribution.

The main estimating equations for the analyses were multilevel logistic regression models using random intercepts at the mother level:

$$\Pr\left( y_{ij}=1 \right)=F(\beta_{0}+ \beta_{n}x_{nij}+ \mu_{j})$$

Where $i$ refers to birth (1, …, *n*) for mother $j$, $y$ refers to the outcome (e.g. infant mortality), $\beta_{0}$ refers to the constant, and $x_{n}$ refers to infant- mother- and household-level variables with their respective coefficients $\beta_{n}$. $\mu_{j}$ refers to the error term for each mother. *F(z) = e^z^ / (1 + e^z^)* is the cumulative logistic distribution.

These equations operationalised in STATA MP statistical analysis software using the logit and melogit commands.

**Table S1 – Logistic regression on likelihood of any maternal FHS utilisation before third trimester of pregnancy**

|  | **aOR** | **95%CI** |
| --- | --- | --- |
| **Mother-level variables** |  |  |
| Maternal age |  |  |
| <17 years | 1 (Ref) |  |
| 18-19 years | 1.006 | 0.911,1.111 |
| 20-24 years | 1.030 | 0.943,1.126 |
| 25-29 years | 1.260*** | 1.147,1.383 |
| 30-34 years | 1.443*** | 1.305,1.594 |
| 35-39 years | 1.479*** | 1.318,1.660 |
| 40-44 years | 1.553*** | 1.306,1.848 |
| 45-50 years | 1.780 | 0.965,3.285 |
| Mother's marital status |  |  |
| Single | 1 (Ref) |  |
| Married/Civil Union | 0.885** | 0.822,0.952 |
| Widow/Separated/Other | 1.033 | 0.856,1.246 |
| Maternal educational attainment |  |  |
| Less than 3 years | 1 (Ref) |  |
| 4-7 years | 1.183* | 1.039,1.346 |
| 8-11 years | 1.693*** | 1.493,1.921 |
| 12+ years | 0.724*** | 0.601,0.872 |
| Maternal race/ethnicity |  |  |
| White | 1 (Ref) |  |
| Black | 1.006 | 0.936,1.081 |
| Parda | 0.998 | 0.943,1.056 |
| Asian or Indigenous | 0.046*** | 0.012,0.176 |
| Mother has other children | 0.804*** | 0.758,0.854 |
| Any of mother's other children have died | 0.777*** | 0.736,0.820 |
| Mother has a disability | 0.822 | 0.657,1.028 |
| Mother is employed | 0.987 | 0.939,1.037 |
| Mother's hospitalisations before pregnancy | 1.927*** | 1.801,2.061 |
| Private hospital birth | 0.438*** | 0.414,0.464 |
|  |  |  |
| **Household-level variables** |  |  |
| Child labour in household? | 1.018 | 0.798,1.299 |
| Household has piped water | 1.215 | 0.999,1.478 |
| Bolsa Familia recipient household | 0.964 | 0.887,1.047 |
| Formal employment in household | 1.068* | 1.001,1.139 |
| Inhabitants per bedroom |  |  |
| less than two | 1 (Ref) |  |
| two to three | 0.914** | 0.859,0.971 |
| three to four | 0.930* | 0.866,0.999 |
| four or more | 0.813*** | 0.752,0.878 |
| Household expenditure on medicines |  |  |
| None | 1 (Ref) |  |
| 0-R$50 per month | 1.060 | 0.989,1.136 |
| More than R$50 per month | 1.023 | 0.911,1.149 |
| Household per capita income quintile |  |  |
| Q1 (lowest) | 1 (Ref) |  |
| Q2 | 1.140*** | 1.057,1.230 |
| Q3 | 1.158*** | 1.071,1.251 |
| Q4 | 1.096* | 1.011,1.188 |
| Q5 (highest) | 1.085 | 0.990,1.189 |
|  |  |  |
| N (Observations) | 74107 |  |

Model also adjusted for cohort entry date and bairro of residence;

**Figure S1 – Inequalities in association between FHS use during first two trimesters of pregnancy and secondary outcomes by socioeconomic groups**

**
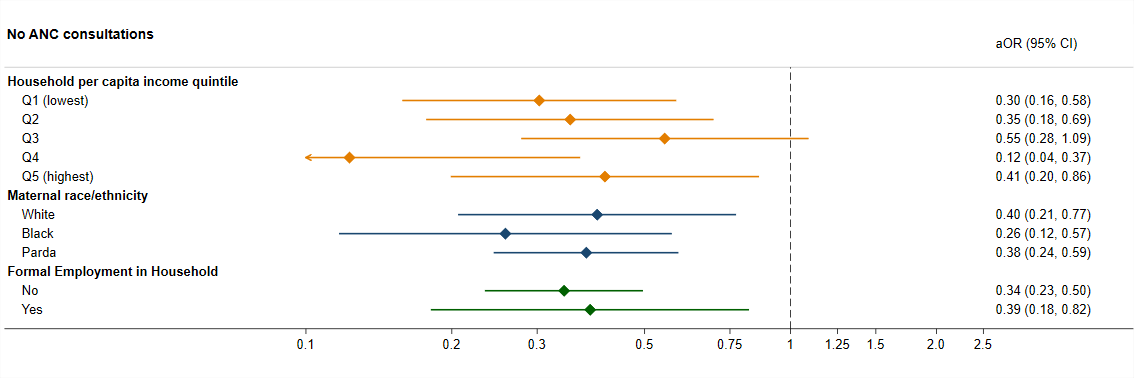

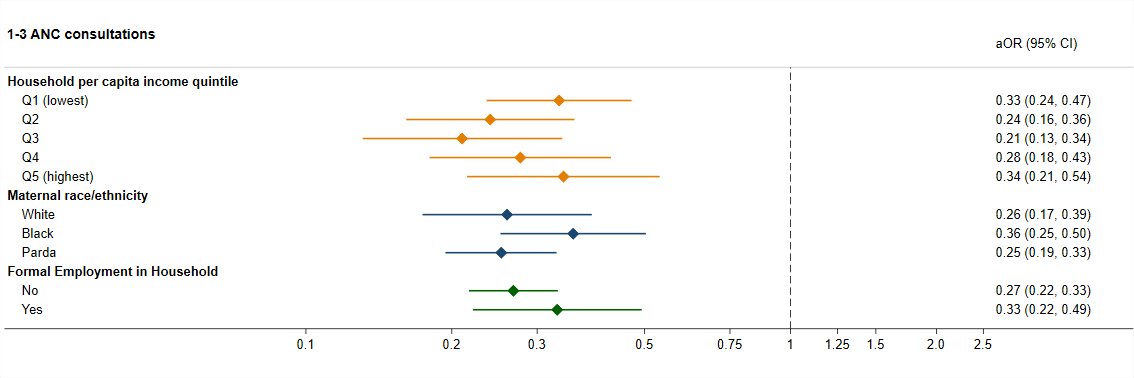

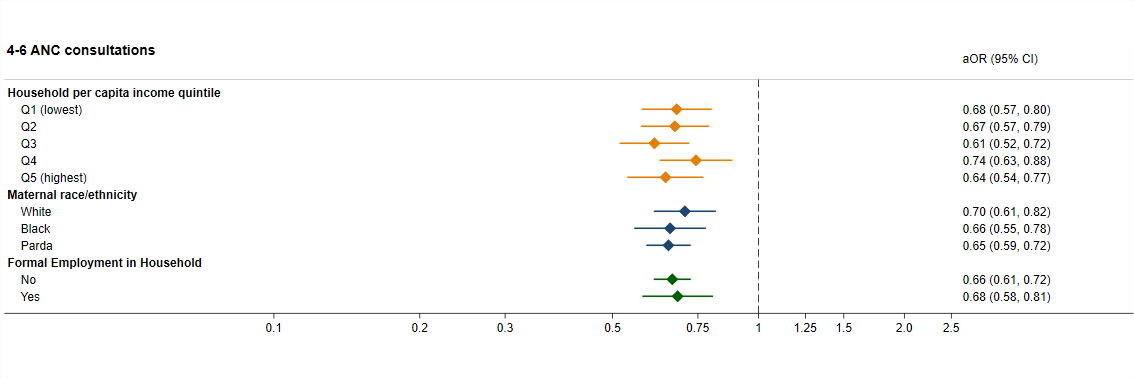

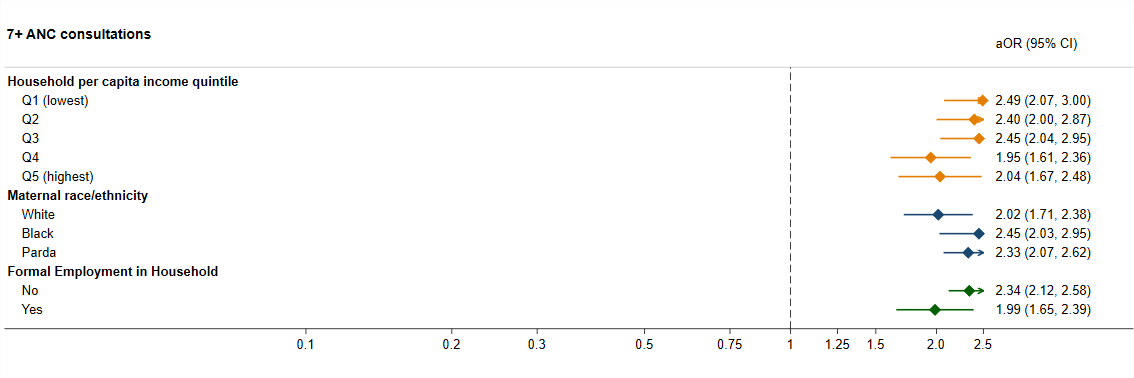
**

**
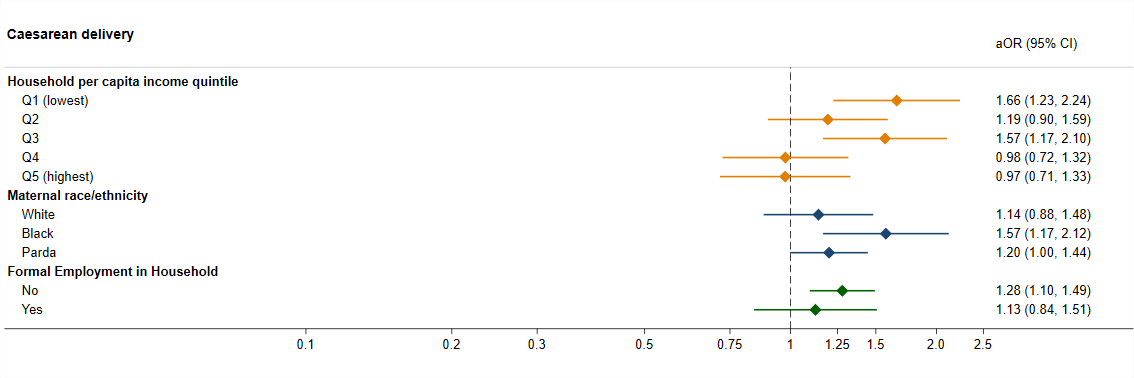

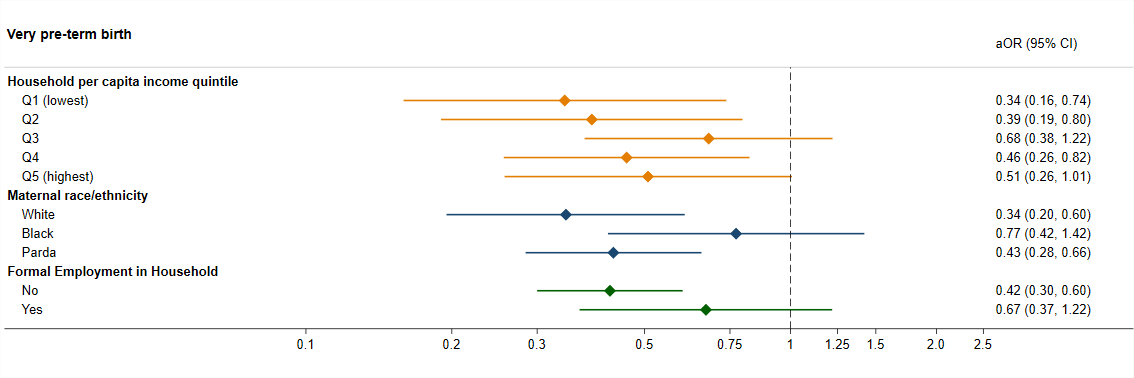

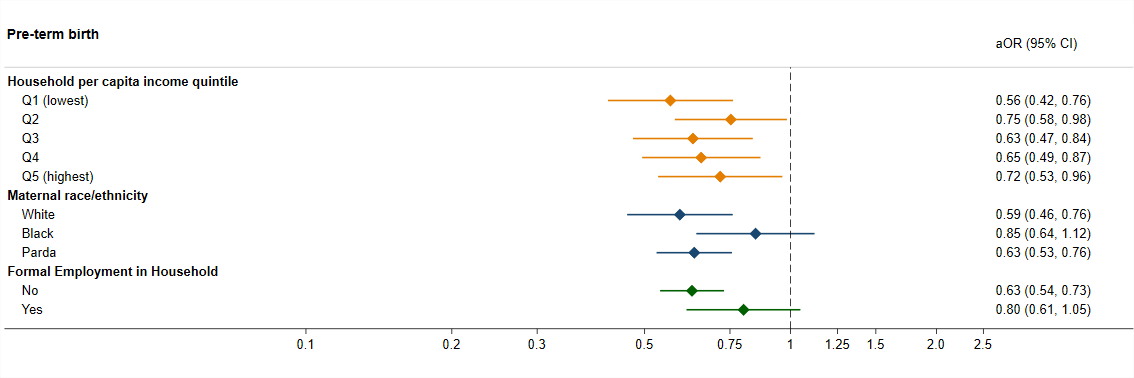

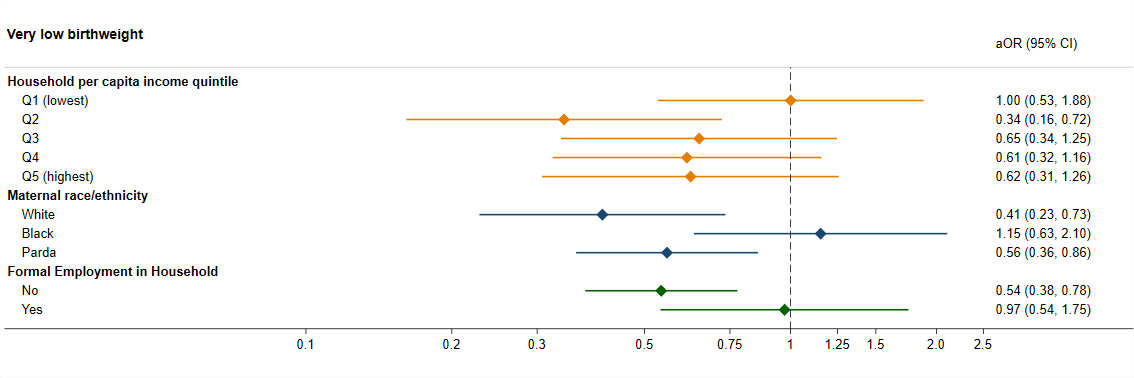

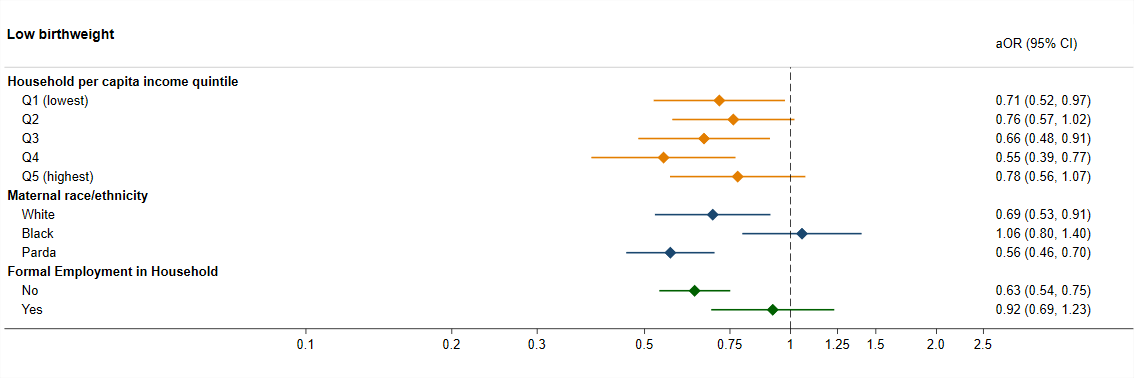
**

**Table S3 – Results from IPTW-RA multilevel logistic regression models on neonatal and infant death, ANC consultations, and birth outcomes (only third trimester FHS use)**

|  | **Any maternal FHS consultation during 3^rd^ trimester** | |
| --- | --- | --- |
|  | **aOR** | **95%CI** |
| **Primary Outcomes** |  |  |
| Neonatal death | 0.845 | 0.590,1.211 |
| Infant death | 0.915 | 0.705,1.188 |
|  |  |  |
| **Secondary Outcomes** |  |  |
| No ANC | 0.233*** | 0.168,0.322 |
| 1-3 ANC cons | 0.529*** | 0.460,0.609 |
| 4-6 ANC cons | 0.829*** | 0.774,0.889 |
| 7+ ANC cons | 1.630*** | 1.508,1.761 |
| Caesarean birth | 0.805*** | 0.711,0.911 |
| Very preterm birth | 0.748* | 0.585,0.958 |
| Preterm birth | 0.859** | 0.765,0.964 |
| Very low birth weight | 0.789 | 0.606,1.028 |
| Low birth weight | 0.790*** | 0.693,0.901 |

Each coefficient is from a separate logistic regression model. All models were adjusted for: month and year of birth; type of birth (single; twins; triplets or more); infant’s sex; mother’s self-identified race/ethnicity; mother’s marital status; mother’s age; mother’s educational attainment; mother’s disability; mother’s employment status; if the mother has other children; if the mother had other children who died; household income quintile; household inhabitants per bedroom; household per capita monthly expenditure on medicine; household formal employment; household Bolsa Familia receipt; household access to water; and if there was child labour in the household. Standard errors clustered by mothers. Models weighted by IPTW.

ANC – Antenatal care; FHS – family health strategy; IPTW-RA – inverse probability of treatment weighting with regression adjustment; aOR – adjusted Odds Ratio; 95%CI – 95% confidence interval; * p < 0.05, ** p < 0.01, *** p < 0.001

**Table S4 – Results from IPTW-RA logistic regression models on neonatal and infant death, ANC consultations, and birth outcomes (including only first birth per mother)**

|  | **Any maternal FHS consultation before pregnancy** | | **Any maternal FHS consultation 1st or 2nd trimester** | |
| --- | --- | --- | --- | --- |
|  | **aOR** | **95%CI** | **aOR** | **95%CI** |
| **Primary Outcomes** |  |  |  |  |
| Neonatal death | 1.256 | 0.737,2.142 | 0.522** | 0.333,0.818 |
| Infant death | 1.079 | 0.698,1.668 | 0.728 | 0.523,1.014 |
|  |  |  |  |  |
| **Secondary Outcomes** |  |  |  |  |
| No ANC | 0.446** | 0.267,0.742 | 0.448*** | 0.318,0.631 |
| 1-3 ANC cons | 0.560*** | 0.433,0.724 | 0.335*** | 0.275,0.408 |
| 4-6 ANC cons | 0.729*** | 0.652,0.816 | 0.726*** | 0.674,0.782 |
| 7+ ANC cons | 1.577*** | 1.416,1.757 | 1.738*** | 1.616,1.868 |
| Caesarean birth | 1.297*** | 1.178,1.428 | 1.123*** | 1.053,1.198 |
| Very preterm birth | 0.853 | 0.588,1.238 | 0.517*** | 0.388,0.690 |
| Preterm birth | 0.920 | 0.792,1.069 | 0.724*** | 0.651,0.805 |
| Very low birth weight | 0.905 | 0.607,1.349 | 0.614** | 0.453,0.831 |
| Low birth weight | 0.937 | 0.790,1.111 | 0.751*** | 0.667,0.845 |

Each coefficient is from a separate logistic regression model, but including on the first birth per mother in the cohort (n= 66034). All models were adjusted for: month and year of birth; type of birth (single; twins; triplets or more); infant’s sex; mother’s self-identified race/ethnicity; mother’s marital status; mother’s age; mother’s educational attainment; mother’s disability; mother’s employment status; if the mother has other children; if the mother had other children who died; household income quintile; household inhabitants per bedroom; household per capita monthly expenditure on medicine; household formal employment; household Bolsa Familia receipt; household access to water; and if there was child labour in the household. Standard errors clustered by mothers. Models weighted by IPTW.

ANC – Antenatal care; FHS – family health strategy; IPTW-RA – inverse probability of treatment weighting with regression adjustment; aOR – adjusted Odds Ratio; 95%CI – 95% confidence interval; * p < 0.05, ** p < 0.01, *** p < 0.001
