# Supplementary material for: Associations between primary healthcare and infant health outcomes: a cohort analysis of low-income mothers in Rio de Janeiro, Brazil
Source: Lancet Reg Health Am. 2023 May 25;22:100519. doi: 10.1016/j.lana.2023.100519 (PMC10238835; doi:10.1016/j.lana.2023.100519)
Supplement: Abstract in portuguese [file mmc2.docx]

***Editorial Disclaimer:*** *This translation in Portuguese was submitted by the authors and we reproduce it as supplied. It has not been peer-reviewed. Our editorial processes have only been applied to the original abstract in English, which should serve as a reference for this manuscript.*

**Resumo**

**Introdução:** A expansão da atenção primária à população urbana pobre é uma prioridade em muitos países de baixa e média renda e é essencial para alcançar a cobertura universal em saúde (CUS). Entre 2008-16, a cidade do Rio de Janeiro empreendeu um ambicioso programa para expandir rapidamente a atenção primária para áreas de baixa renda por meio da Estratégia da Saúde da Família (ESF). Os impactos desta expansão na saúde infantil são desconhecidos. Este estudo examina as associações entre a utilização materna da ESF e os desfechos do parto, mortalidade neonatal e infantil.

**Métodos:** Uma coorte de 75.339 nascidos vivos (janeiro de 2009 a dezembro de 2014) de mães de baixa renda no Rio de Janeiro foi vinculada a registros de atenção primária, nascimento, hospital e óbito. A relação entre o uso da ESF materna e os desfechos de saúde infantil foi avaliada por meio de regressão logística com probabilidade inversa de ponderação de tratamento e ajuste do modelo. Desigualdades socioeconômicas nas associações entre uso da ESF e desfechos foram exploradas por meio de interações. Os desfechos primários foram óbitos neonatais e infantis. Treze desfechos secundários também foram examinados para explorar outros desfechos de saúde importantes e seus potenciais mecanismos.

**Resultados:** Um total de 9.002 (12,0%) bebês nasceram de mães da coorte que usaram serviços da ESF antes da gravidez ou nos dois primeiros trimestres. Houve 527 óbitos neonatais e 893 infantis. O uso de ESF materna durante os dois primeiros trimestres foi associado a reduções substanciais na mortalidade neonatal [razão das chances ajustada (aOR): 0,527, intervalo de confiança de 95% (IC 95%): 0,345;0,806] e mortalidade infantil (aOR: 0,672, IC 95% : 0,48;0,924). Crianças nascidas de mães de baixa renda e aquelas sem emprego formal tiveram maiores reduções na mortalidade neonatal e infantil associadas ao uso da ESF. O uso da ESF materna nos dois primeiros trimestres também foi associado a mais consultas de pré-natal e a um menor risco de baixo peso ao nascer e de parto prematuro.

**Interpretação:** A expansão da atenção primária para populações de baixa renda no Rio de Janeiro foi associada a benefícios de saúde infantil e à equidade em saúde.

**Financiamento:** DFID/MRC/Wellcome Trust/ESRC
